# Network Biology of Alzheimer’s Disease and Related Neurodegenerative Disorders: Molecular Mechanisms and Therapeutic Strategies

**DOI:** 10.3390/biom16070944

**Published:** 2026-06-24

**Authors:** Zitin Wali, Neha, Moyad Shahwan, Khuzin Dinislam, Anas Shamsi, Saleha Anwar

**Affiliations:** 1 Department of Toxicology, School of Chemical and Life Sciences, Jamia Hamdard, New Delhi 110062, Delhi, India; zitinwali@gmail.com (Z.W.); email2saleha@gmail.com (S.A.); 2 Department of Forensic Science, School of Basic & Applied Sciences, SGT University, Gurugram 122505, Haryana, India; 3 Department of Clinical Sciences, College of Pharmacy and Health Sciences, Ajman University, Ajman 346, United Arab Emirates; m.shahwan@ajman.ac.ae; 4 Center of Excellence in Precision Medicine and Digital Health, Chulalongkorn University, Bangkok 10330, Thailand; 5 Department of General Chemistry, Bashkir State Medical University, Ulitsa Lenina, 3, 450008 Ufa, Republic of Bashkortostan, Russia; dinis.d.2012@mail.ru; 6 Center of Medical and Bio-Allied Health Sciences Research, Ajman University, Ajman 346, United Arab Emirates

**Keywords:** therapeutic strategies, neurodegenerative diseases, neuroinflammation, pathophysiology, genetics

## Abstract

The most persistent biomedical challenges of the 21st century are neurodegenerative disorders (NDs), where molecular alterations lead to devastating clinical consequences and progressive neuronal loss. The prevalence of neurodegeneration is continuously rising and becoming the main contributor to chronic disability and mortality. Despite their clinical differences, many conditions share pathogenic processes, including oxidative stress, protein misfolding and aggregation, mitochondrial dysfunction, and neuroinflammation. Instead of functioning independently, these processes cooperate to form a self-reinforcing network that gradually weakens synapses and ultimately leads to neuronal death. This study redefines neurodegeneration as a disorder of system-level failure by emphasizing poor cellular stress integration. In addition to demonstrating how gut microbiome gene networks impact inflammation and amyloid production, new research highlights the relationships between mitochondrial–lysosomal interactions, endoplasmic reticulum stress responses, and transcriptionally driven synaptic vulnerability. A key molecular topic is the interaction and pathogenic convergence of the JAK/STAT, HIF-1α, and Notch signaling pathways. Under ongoing metabolic stress, prolonged stimulation of this triad increases inflammation, hinders the regenerative processes, and maintains pseudo-hypoxic conditions, explaining why single-target treatments have mostly been unsuccessful. This review also explores progress in fluid, digital, and imaging biomarkers that facilitate early diagnosis and patient stratification, and assesses new disease-modifying approaches such as antisense oligonucleotides, immunomodulators, gene therapies, and small-molecular agents. Artificial intelligence is emphasized as an essential tool for integrating multimodal data, drug discovery and predictive modeling.

## 1. Introduction

Neurodegenerative diseases (NDs) involve a relentless loss of neurons, leading to serious neurological symptoms and often death. As people around the world get older, these age-related brain diseases are becoming more common. Alzheimer’s disease (AD) is the predominant cause of dementia, accounting for approximately 60–70% of all cases worldwide. As of now, about 57 million people worldwide live with dementia, and each year another 10 million cases are diagnosed. In the United States alone, 6.9 million people aged 65 and above suffer from AD. By 2050, experts expect that number to nearly double. We also highlight amyotrophic lateral sclerosis (ALS), a unique motor neuron disease, even though dementia and AD predominate within the spectrum of NDs. Depending on the population and location, ALS has a global point prevalence of ~1.6 to 11.8 per 100,000 and an incidence of ~0.3 to 23 per 100,000 person-years [1]. According to estimates, the prevalence of ALS in the United States was ~9.9 cases per 100,000 in 2022 (32,893 cases), and by 2030, it is expected to have increased by more than 10% [2,3]. Similarly, Huntington’s disease (HD), an autosomal dominant condition brought on by the expansion of the CAG repeat in the HTT gene, affects ~2.71 out of every 100,000 people globally, with a higher incidence of 0.38 per 100,000 per year in Europe, North America, and Australia than in Asia and Africa [4]. Approximately 2.8 million individuals globally are affected by multiple sclerosis (MS), and its prevalence varies greatly by latitude and location [5]. One of the recent findings states that the major cause of non-traumatic neurological disturbances in young individuals is MS [6]. Epidemiological statistics show that NDs have a huge impact on public health, and it is crucial to understand both their common and disease-specific causes [7].

The aggregation and misfolding of harmful proteins are prevalent characteristics of NDs. These include Alzheimer’s disease (AD), which is characterized by amyloid-β plaques and hyperphosphorylated tau, while Parkinson’s disease (PD) features α-synuclein aggregates, ALS presents with aggregated protein “TDP-43” and misfolded proteins, and Huntington’s disease (HD) exhibits an expanded polyglutamine tract in mutant huntingtin (mHTT). These varied entities and their altered structures, with glial activation, initiate the mechanisms of oxidative stress, mitochondrial dysfunction, dysregulation of lysosomal activity, and increased excitotoxicity, triggering neuroimmune imbalance that ultimately contributes to the pathogenesis of neurodegenerative disorders [8,9,10]. Numerous articles state that targeting integrated approaches in signaling mechanisms, including JAK-STAT, HIF-1 α, and Notch, opens up therapeutic potential by regulating glial activation, neuroinflammation, hypoxia and cellular metabolism [11,12,13,14,15].

Even after decades of research, millions of people worldwide continue to experience progressive loss of memory, mobility, and independence. Existing therapies, such as dopaminergic medications, cholinesterase inhibitors, and riluzole, only provide temporary symptom alleviation [16,17,18]. As a result, scientists are working to create therapies that go beyond symptom relief and address the underlying causes of the condition. Existing methods aim to address the source of damage by promoting mitochondrial repair, reducing proteotoxic stress, enhancing the clearance of cellular waste, and restoring immune–brain interactions. Small molecules that help maintain redox homeostasis and mitochondrial function, agents that impact synaptic and inhibitory signaling, monoclonal antibodies against misfolded proteins, immune-based treatments, and strategies to fortify the blood–brain barrier (BBB) while encouraging the removal of dangerous substances from the brain are all being investigated in current research [19,20,21,22]. Blending fluid, imaging and digital biomarker discovery allows early disease diagnosis, categorizes patients more reliably and tracks their progress in real time [23,24]. Researchers depend heavily on computational tools, systems biology, and artificial intelligence (AI) to accelerate target identification, drug repurposing, and disease prediction [20,25]. In this review, mechanistic insights from different NDs, connecting proteopathy, autophagy, mitochondrial and neuroimmune dysfunctions, are brought together into a unified perspective on disease progression. The current article also highlights the importance of AI in drug development, computational simulations, systems biology and biomarker-guided personalized medicine.

## 2. Pathophysiological Mechanisms

### 2.1. Protein Misfolding and Aggregation

**Figure 1:**
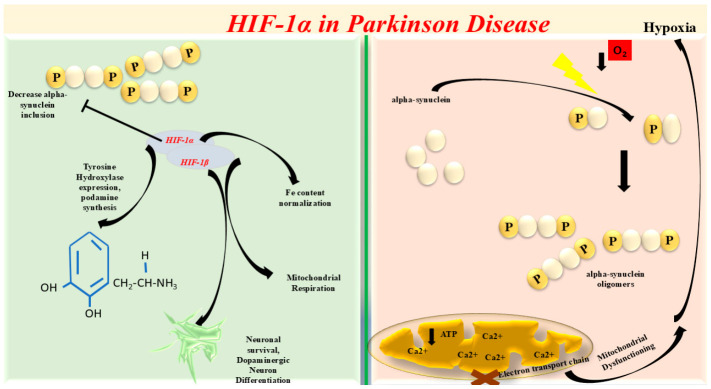
Role of HIF-1α in Parkinson’s Disease Pathogenesis. Schematic representation illustrating the involvement of hypoxia-inducible factor-1 alpha (HIF-1α) in Parkinson’s disease. Under normoxic conditions, HIF-1α is hydroxylated and degraded, maintaining cellular homeostasis. However, under hypoxic conditions, stabilization of HIF-1α leads to increased α-synuclein aggregation and mitochondrial dysfunction. This process disrupts oxidative phosphorylation, reduces ATP production, and elevates intracellular Ca^2+^ levels, ultimately contributing to dopaminergic neuronal degeneration. Additionally, impaired tyrosine hydroxylase activity affects dopamine synthesis, further exacerbating neurodegeneration. This figure was created in Microsoft PowerPoint.

Protein misfolding occurs at the molecular level when native structures are disrupted, revealing β-sheet-prone and hydrophobic regions that are typically hidden within the folded structure. This conformational plasticity enables aberrant intermolecular contacts, starting a nucleation-driven aggregation cascade that produces soluble oligomers and, ultimately, insoluble fibrils. In AD, the α-helical or random coil forms of Aβ peptides are replaced by cross-β-sheet topologies, which self-template into polymorphic fibrils with unique hazardous “strains” [26]. In PD, α-synuclein is the primary pathogenic driver (Figure 1) and its intrinsic abnormality allows for membrane-induced conformational changes and secondary nucleation on pre-existing fibril surfaces, producing extremely toxic oligomeric species [27]. As seen by the co-assembly of Aβ and α-synuclein, which results in hybrid oligomers with changed dynamics and increased neurotoxicity, cross-seeding further intensifies disease [28]. Low-complexity protein domains, such as those in TDP-43, promote liquid–liquid phase separation in ALS and related proteinopathies, raising local protein concentrations and reducing energy barriers to permanent aggregation [29]. Chemical reactions inside the body of individuals can make some proteins unstable, causing them to change shape and size, generating toxic derivatives and disturbing the normal cycle of proteins inside the cells (proteostasis). This process is regulated by sequences of proteins and how they naturally fold, post-translational modifications and the environment around the cells. When the proteins fold incorrectly, they produce a small toxic group termed oligomers, which damage cell membranes and organelles and spread damage from one area to another [30,31].

One intriguing treatment approach is to use small molecules or molecular chaperones to maintain proper protein folding and prevent aggregation. Molecular chaperones are highly specific proteins that help maintain proteostasis by preventing aberrant intermolecular interactions, facilitating the refolding or degradation of misfolded proteins, and assisting nascent or stress-denatured polypeptides in attaining and retaining their native conformations (see Table 1 for representative chaperone systems and their mechanisms of action). Pharmacological chaperones stabilize weak protein conformers to replicate this action. Tafamidis, which interacts with transthyretin (TTR) tetramers and stabilizes their quaternary structure by inhibiting dissociation into aggregation-prone monomers, is a clinically verified example. Clinical research shows that tafamidis significantly lowers the production of harmful amyloid fibrils linked to transthyretin amyloid cardiomyopathy (ATTR-CM) by stabilizing both wild-type and mutant TTR [32]. Another treatment option is to develop substances that prevent the interactions that drive protein aggregation. Recently, small compounds that prevent the aggregation of proteins like tau and α-synuclein, linked to AD and PD, respectively, have been discovered. By stopping the production of harmful oligomers and fibrils, these inhibitors can slow the advancement of disease [30]. Researchers are exploring antibodies to eliminate misfolded proteins, a viable method to treat protein misfolding disorders. By engineering monoclonal antibodies that specifically recognize and bind misfolded protein conformations, researchers can facilitate immune-mediated clearance of these pathogenic aggregates. Several clinical trials are currently evaluating the therapeutic potential of this strategy across various NDs [31].

### 2.2. Genetic Aspects of Cellular Networks

Science and its mechanisms are interdependent; for example, mitochondria provide energy while lysosomes handle mitophagy and autophagy. Our body system requires energy to regulate calcium ion flow and balance homeostasis. Mitochondria are the friends of lysosomes, providing energy and sending signals to help lysosomes work properly. If there is any mutation or dysregulation within this integrated system, the whole mechanism reverses and drives the convergent molecular axis in NDs [43,44]. This functionally coordinated interaction is not only superficial but also deep-rooted, where disturbances at the gene level, such as ATP depletion, excessive ROS production, and calcium ion dysregulation, are directly linked to mitochondrial dynamics, the electron transport chain and autophagy in NDs [45,46]. Glucocerebroside is an enzyme encoded by the GBA gene in lysosomes, and mutations in GBA directly affect the ND genetic axis of vulnerability [47]. This association established the proof-of-concept that mitochondria–lysosomes are a pharmacological target for treating NDs [48]. Traditional therapeutic approaches were heavily focused on symptomatic treatments (such as tremors and memory loss). Nowadays, scientists are trying to fix the problem inside the cells or the organelles by targeting gene-based and RNA-based therapies [49,50,51]. The process of cleaning and recycling cell waste (autophagy) and its balance are controlled by genes (Endoplasmic Reticulum) and are very crucial for maintaining neurons healthy and for their survival [52]. Neuronal proteostasis depends on the coordinated activity of autophagy-related genes (ATG5, ATG7, BECN1, and LC3) and endoplasmic reticulum (ER) stress sensors, including PERK (EIF2AK3), IRE1 (ERN1), and ATF6. Dysregulation or mutation of these genes can impair protein quality control mechanisms, thereby contributing to the development and progression of NDs [53,54]. The pathways or the signals/genes associated with the ER directly control autophagy at the pre- and post-transcriptional stages by linking mRNA translation to degradation capacity [55]. In NDs, prolonged stress leads to sustained UPR activation, triggering cell death mechanisms. This results in impaired neuronal communication and the accumulation of toxic proteins such as Aβ, tau, α-synuclein, and TDP-43 [56,57,58]. ER stress regulates autophagic flux via the ATF6, PERK-eIF2α, and IRE1-XBP1 pathways, initially boosting the transcription of autophagy-related genes but eventually leading to translational repression and autophagy depletion during prolonged stress [59]. In ALS, C9ORF72 mutations disrupt autophagy initiation by affecting mRNA stability and vesicle transport, whereas in AD, ongoing UPR signaling promotes tau hyperphosphorylation through stress-activated kinases. ER-mitochondria junctions serve functions beyond linking two organelles; they govern calcium-driven transcriptional activity and regulate genes responsible for mitophagy. Intimate interactions among organelles are crucial for disease progression [45,60]. Due to these insights, researchers now focus on gene- and RNA-based therapies. Instead of blocking ER signaling altogether, UPR regulators and drug-based chaperones modify stress-activated transcriptional programs to restore balance [61]. Additional attempts focus on autophagy. Researchers employ activators independent of mTOR, AMPK signaling modulators, and RNA-mediated regulators for upregulating the expression or translation of lysosomal and autophagy-related genes [43,59].

There is growing evidence that synaptic loss and neuron death in NDs do not occur by chance; they are strongly linked to genetics. When the genes regulating synaptic function, metabolism and cell death lose their balance, the entire system suffers. Neurons rely on precise transcription of synaptic genes to remain linked. These include genes for presynaptic vesicle proteins (SNAP25, SYN1), postsynaptic scaffolding proteins (PSD95/DLG4), glutamate receptors (GRIN1/2) and calcium regulators (CACNA1C, ATP2B). In AD, Aβ and tau interfere with genetic pathways responsible for synaptic health. This disruption begins early, leading to decreased expression of key mRNAs linked to plasticity and activity-driven genes, well before visible neuron loss [62,63]. In PD and HD, mutations in SNCA, LRRK2, and HTT perturb vesicle trafficking, axonal transport, and the transcriptional regulation of synaptic proteins, destabilizing neuronal networks [64,65]. Neuronal death reflects the activation of genetically encoded cell-death programs. OS and calcium dysregulation induce the expression of pro-apoptotic genes (BAX, PUMA, CASP3), while suppressing survival pathways (BDNF, CREB) [66]. In AD, excessive NMDA receptor signaling upregulates calcium-responsive death pathways, whereas in PD, α-synuclein–driven transcriptional stress converges on mitochondrial apoptotic cascades [65,67]. Significantly, repression of synaptic genes occurs before the activation of death-related mRNAs, establishing synaptic failure as a transcriptionally prepared pathway to neuronal death. This genetic continuum reshapes therapy to focus on maintaining synaptic transcriptomes and inhibiting harmful death mechanisms. Modulating plasticity genes, stress-responsive transcription factors and calcium-handling mechanisms through RNA-targeting provides a logical approach to fortify circuits and postpone irreversible neuronal loss in NDs.

Neuroinflammation in NDs is increasingly recognized as a genetically orchestrated mechanism influenced by imbalances in immune gene networks within peripheral immune cells, microglia, and astrocytes. Transcriptomic and genome-wide investigations uncover both disease-specific and common stimulation of innate immune genes, such as TNF, TREM2, IL1B, and APOE, that influence the inflammatory profile and microglial phenotype [68,69]. In AD, TREM2 and APOE alter microglia transcription, shifting toward a disease-related state that promotes ongoing cytokine production and synaptic pruning. In HD, prolonged upregulation of TNF and IL6 speeds up neuronal impairment and network failure [70,71]. Aging exacerbates these transcriptional changes via epigenetic drift and weakened immune gene repression, leading to a lasting “inflammaging” profile [72]. Dysregulation of the adaptive immune system is reflected in changes in the expression of regulatory T-cell genes. Decreased FOXP3 activity and regulatory T-cell-related transcripts, combined with increased effector T-cell gene expression, shift the immune equilibrium towards neurotoxicity in ALS, PD and AD [73]. Misfolded proteins serve as immunogenic templates, promoting the expression of genes with antigens and autoimmune-like responses [74]. Gut microbiome dysbiosis likewise modifies peripheral immune transcriptomes, leading to neuroimmune communication and synaptic inflammation that impairs protein clearance [75]. Hence, neurodegeneration is more often recognized as a disorder involving immune gene regulation, shifting treatment focus towards the transcriptomic reprogramming of T-cells and microglia to reinstate neuroprotective conditions and mitigate inflammation.

### 2.3. Gut Microbial Gene Networks

A gut microbiome represents a genetically diverse ecosystem, and its collective genome plays a crucial role in influencing host neurology. Metagenomic investigations of NDs are progressively showing that disease-associated dysbiosis is functional rather than just compositional, characterized by modified microbial gene repertoires controlling immunological regulation, metabolism, and the manufacture of neuroactive compounds [76,77]. By producing bile acids, neurotransmitter precursors, short-chain fatty acids (SCFAs), and inflammatory mediators, these microbial genes influence the microbiota–gut–brain axis and connect gut ecology to central nervous system homeostasis [78,79]. In PD, studies show fewer gut bacteria that produce butyrate, and some crucial genes that reduce the production of short-chain fatty acids (SCFA). This is why the gut barrier becomes weak, and inflammation is very high in the body and brain [80,81]. At the same time, harmful bacterial genes increase and produce lipopolysaccharides and bacterial amyloids, which cause more inflammation inside the cells, triggering the misfolding of a protein (α-synuclein) and worsening the disease by moving from the gut to the brain [82,83]. In AD, an imbalance of gut bacteria (dysbiosis) alters how microbes process important chemicals, including tryptophan, phenylalanine biosynthesis, and γ-aminobutyric acid (GABA) production. This affects the brain signaling system (like serotonin and glutamate) alters microglial responses, and disrupts glutamatergic signaling [76,77].

Metagenomic studies reveal an increased representation of genes encoding pro-inflammatory pathways, including flagellar assembly and peptidoglycan synthesis, which correlate with elevated circulating cytokines and BBB permeability [78,82]. Likewise, microbial genome profiles linked to xenobiotic metabolism and OS responses are abundant in HD and ALS, indicating adaptive microbial mechanisms that worsen host redox dysfunction and mitochondrial sensitivity [80,83]. These findings support the concept that neurodegeneration is influenced by a “second genome” whose transcriptional output continuously interfaces with host neuronal and immune pathways.

The production of SCFAs, particularly butyrate, propionate, and acetate, is controlled by a highly significant class of microbial genes that also control histone acetylation, microglial maturation, and the integrity of the BBB. When these gene clusters are lost, pro-inflammatory microglial phenotypes are preferred, while epigenetic support for neuronal resilience is reduced [79,84]. On the other hand, the growth of genes that encode bacterial amyloids, like curli and Fap, promotes the cross-seeding of host amyloidogenic proteins, such as Aβ and α-synuclein, speeding up the kinetics of aggregation and neurotoxicity [82,83]. This molecular mimicry enables a mechanistic link between proteinopathy and microbial genomes.

Translationally, metagenomic sequencing of disease-specific microbial gene signatures allows for the discovery of therapeutic targets and precise patient classification. It is possible to reintroduce SCFA-producing gene clusters, suppress pro-inflammatory microbial programs, and restore depleted metabolic pathways through the use of engineered probiotics, postbiotics, and dietary treatments [76,84]. More ambitiously, CRISPR-based antimicrobials or bacteriophages could be used in microbiome editing techniques to specifically eradicate pathogenic gene networks while maintaining commensal function [83]. As a result, neurodegeneration is now more widely recognized as a systems-level pathology influenced by the gut microbiome’s genetic output rather than just a problem of the neuronal genome, which is expanding the range of potential treatments along the microbiota–brain continuum.

## 3. Key Signaling Pathways in Neurodegeneration-Targeting HIF-1α–Notch–JAK-STAT Crosstalk

ND are being regarded as maladaptive stress integration diseases rather than separate biological abnormalities. HIF-1α, Notch, and JAK/STAT form a signaling trio that regulates metabolic stress, cell fate, and immune activation. Under physiological circumstances, these pathways regulate neuronal homeostasis; however, in NDs, dysregulation of mitochondria and generated oxidative stress leads to persistent activation of HIF-1α, shifting it from a protective regulator to a chronic stress mediator [85,86]. HIF-1α activation shifts cellular metabolism to glycolysis, enhances STAT3 signaling, and modulates Notch activity, thereby coupling metabolic imbalance with immune dysregulation and altered cell fate decisions [87].

**Figure 2:**
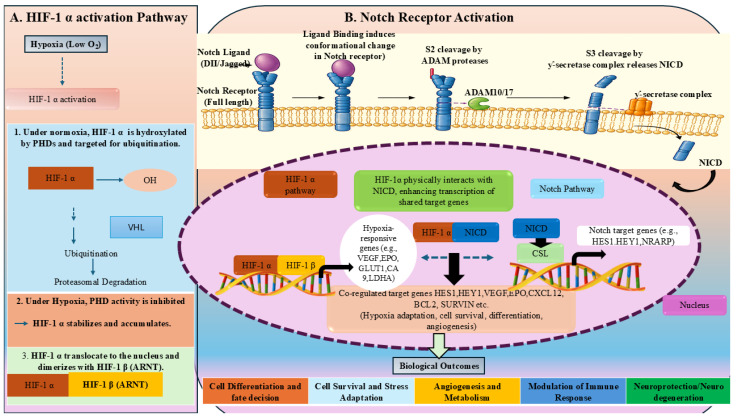
Crosstalk Between HIF-1α and Notch Signaling Pathway. Under hypoxic conditions, HIF-1α is stabilized due to inhibition of PHD-mediated degradation and translocates to the nucleus after dimerization with HIF-1β (ARNT), where it regulates hypoxia-responsive genes. Simultaneously, binding of Delta-like (Dll) or Jagged ligands to the Notch receptor induces sequential proteolytic cleavages by ADAM10/17 and the γ-secretase complex, resulting in the release of the Notch intracellular domain (NICD). NICD translocates to the nucleus and interacts with CSL to activate Notch target genes, including HES1 and HEY1. Nuclear interaction between HIF-1α and NICD enhances transcription of shared target genes involved in hypoxic adaptation, cell survival, angiogenesis, immune regulation, and cell fate determination. Solid arrows indicate activation, dashed arrows represent translocation, red dashed arrows denote hypoxia-induced effects, and curved arrows indicate proteolytic release.

This cross talk becomes maladaptive: JAK/STAT-driven inflammation suppresses regenerative Notch signaling, while Notch dysregulation impairs neurogenesis and synaptic stability. Simultaneously, STAT3-Notch interactions reprogram glial responses toward gliosis rather than repair. The result is a self-amplifying loop in which metabolic stress, inflammation, and defective proteostasis reinforce each other, promoting protein aggregation, synaptic dysfunction, and neuronal loss [88,89]. Recent research indicates that HIF-1α promotes Th17 polarization and STAT3 phosphorylation, suggesting a link between adaptive immune dysregulation and metabolic stress [90]. Simultaneously, HIF-1α modulates ligand sensitivity and chromatin accessibility in the Notch axis, thereby affecting NICD-driven transcription in glial cells and neural progenitors (Figure 2) [91,92]. Accordingly, HIF-1α is positioned as an essential upstream regulator that transforms metabolic disruption into permanent transcriptional modification of the immunological pathway. Its chronic stimulation in AD promotes amyloidogenic processing and Aβ buildup [93], while transitory stimulation promotes neuronal survival through VEGF and erythropoietin production [94,95]. In PD, HIF-1α-driven metabolic rewiring protects dopaminergic neurons against mitochondrial toxins [96], but prolonged activation promotes ROS accumulation and ferroptotic death via STAT3/HIF-1α coupling. HIF-1α’s temporal patterns therefore determine whether it acts as a pathogenic driver or a survival factor.

**Figure 3:**
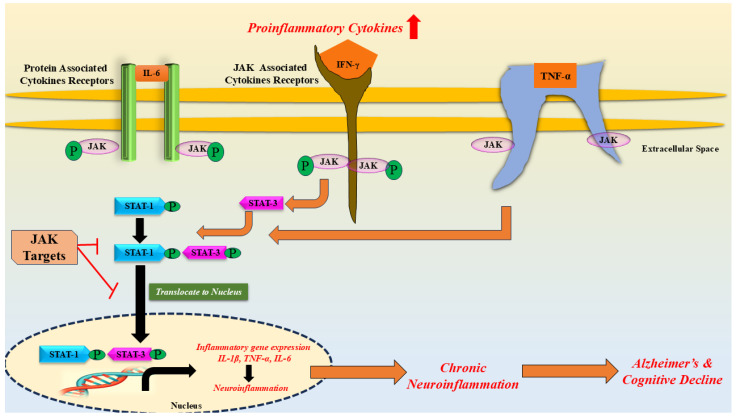
JAK/STAT Signaling in Neuroinflammation and Neurodegeneration. Diagram illustrates the activation of the JAK/STAT signaling pathway in response to pro-inflammatory cytokines such as IL-6, IFN-γ, and TNF-α. Binding of cytokines to their respective receptors activates Janus kinases (JAKs), leading to phosphorylation and dimerization of STAT1 and STAT3. These activated STATs translocate to the nucleus, promoting transcription of inflammatory genes, including IL-6 and TNF-α. Sustained activation of this pathway contributes to chronic neuroinflammation, neuronal damage, and cognitive decline associated with neurodegenerative disorders. This figure was created in Microsoft PowerPoint.

Traditionally thought of as a developmental process, Notch signaling plays a key role in this stress-driven reprogramming. Notch controls lineage selection stabilizes synaptic structure and preserves NSC quiescence in the adult brain [97,98]. Nevertheless, JAK-STAT-mediated cytokine signaling modifies NICD stability, Notch receptor expression and subsequent transcriptional programs under chronic inflammatory and hypoxic circumstances (Figure 3) [13,99]. Via γ-secretase, similar protease complexes that generate APP, Notch is mechanistically connected to amyloidogenesis in AD [100,101]. Early synaptic degeneration and cognitive impairment are influenced by Notch-dependent transcriptional alterations in synaptic gene circuits [102]. In PD, reduced Notch activity compromises adult neurogenesis and dopaminergic replenishment, while inflammatory suppression of Notch in neurogenic niches accelerates neuronal attrition [103,104]. While its suppression restores the ability to repair, prolonged Notch activation in MS prevents remyelination and oligodendrocyte differentiation [105].

This network’s inflammatory foundation is formed by JAK/STAT signaling. A feed-forward cycle of cytokine production, OS and synaptic toxicity is maintained by persistent stimulation of JAK1/STAT3 in astrocytes and microglia [106,107]. When STAT3 builds up surrounding Aβ in AD, it promotes astrogliosis and inhibits aggregate elimination [108,109]. Pharmacological suppression maintains neuronal integrity in PD, while α-synuclein triggers microglial JAK/STAT, intensifying dopaminergic neuron loss [110]. Interestingly, accumulating evidence suggests that Aβ pathology may also contribute to dopaminergic neurodegeneration in PD, indicating a potential mechanistic convergence between AD- and PD-associated signaling pathways. For example, Aβ accumulation has been linked to the loss of dopaminergic neurons and may exacerbate neuroinflammatory and neurodegenerative processes in PD [111]. Significantly, by distorting the NSC pathway and blocking regenerative processes, JAK/STAT signaling inhibits neurogenic potential [112]. STAT3 rewires glial and progenitor gene expression by interacting with Notch, which causes repair processes to shift in favor of gliosis and neurodegeneration.

The pathological convergence of these pathways generates a self-sustaining network: mitochondrial dysfunction stabilizes HIF-1α; HIF-1α amplifies STAT3 and modulates Notch; JAK/STAT-driven inflammation suppresses reparative Notch signaling; Notch dysregulation impairs neurogenesis and synaptic maintenance; and proteotoxic stress further damages mitochondria, perpetuating pseudo-hypoxia. This feed-forward process explains how neurodegeneration persists even after the first trigger has been eliminated and why different diseases share characteristics of immunological dysfunction, metabolic breakdown and synaptic dysfunction. The junction of Notch-directed suppression of oligodendrocyte differentiation, JAK/STAT-mediated immune invasion and HIF-1α-regulated ROS links hypoxia, inflammation and failed remyelination in MS [105,113]. In HD and vascular dementia, aberrant HIF-1α and Notch signaling destabilize mitochondrial networks and synaptic architecture under chronic hypoperfusion [91,114].

## 4. Diagnosis and Therapeutic Strategies

### 4.1. Biomarker and Diagnostic Techniques

Historically, the primary methods for diagnosing neurodegenerative disorders (NDs) were clinical examination and neuroimaging. Emerging biomarkers enable earlier and more accurate diagnosis, as well as individualized therapeutic techniques, providing measurable and objective indicators of disease progression, molecular pathology, and patient response to therapy [115,116]. By providing disease-specific fingerprints and possible treatment targets, proteomic and metabolomic analyses broaden the search for biomarkers [117,118]. Recent advances have identified microtubule-binding region tau fragment 243 (MTBR-tau243) as a promising fluid biomarker for Alzheimer’s disease. MTBR-tau243, detectable in cerebrospinal fluid and increasingly explored in plasma, exhibits a strong correlation with the burden of insoluble neurofibrillary tau tangles, thereby providing a direct measure of tau pathology. Owing to its high specificity for tau aggregation, MTBR-tau243 has emerged as a valuable candidate for disease staging, monitoring progression, and evaluating therapeutic responses in AD [119].

With unique expression patterns associated with NDs, microRNAs (miRNAs) have become attractive non-invasive biomarkers [120]. miRNAs offer both diagnostic and mechanistic insights through their regulatory roles in synaptic plasticity and apoptosis. By enabling continuous, real-world disease surveillance, digital biomarkers obtained from wearables, voice analytics and behavioral monitoring supplement traditional assessments [121]. While network-based MRI techniques aid in linking immune-mediated damage to NDs like MS, neuroimaging modalities such as PET and MRI remain essential [122]. Because biomarker panels accurately reflect disease heterogeneity and facilitate patient stratification for personalized medicine and clinical trials, they are widely preferred over single markers [123,124]. 

### 4.2. FDA-Approved Treatments

Therapeutic developments in NDs have been profoundly impacted by FDA approvals, which have caused clinical practice to shift from symptom control to disease-modifying approaches. Anti-amyloid monoclonal antibodies like aducanumab and lecanemab have refocused attention on targeting core pathology in AD, whereas cholinesterase inhibitors (donepezil, rivastigmine, galantamine) and the NMDA receptor antagonist memantine alleviate symptoms [125,126]. Levodopa/carbidopa and dopamine agonists are the first-line therapy for PD, with adjuvant medications like COMT and MAO-B inhibitors serving as a backup. Recent studies using agonists of the GLP-1 receptor point to metabolic regulation as a possible neuroprotective approach [127,128].

Immunomodulators that target B-cell depletion, which include interferon-β, glatiramer acetate, sphingosine-1-phosphate (S1P) modulators (fingolimod, siponimod), and monoclonal antibodies like ocrelizumab, are the mainstays of MS treatments [129]. Riluzole and edaravone remain licensed for ALS, whereas sodium phenylbutyrate/taurursodiol (Relyvrio) is becoming more well-known for its ability to target ER and mitochondrial stress pathways [130].

Clinical trial designs that prioritize biomarkers, stratification, and early interventions have been sparked by the FDA’s changing regulatory posture, especially with the approval of aducanumab and lecanemab [131,132]. Table 2 summarizes the FDA-approved therapies for major NDs and their corresponding molecular targets and mechanisms of action.

### 4.3. Emerging Therapies and Clinical Trials

In NDs, the emphasis of treatment is moving from symptom reduction to disease management and repair. Antisense oligonucleotides (ASOs), gene therapy, immunotherapy and small molecule regulators of protein aggregation are examples of emerging methods. With studies showing neural longevity and synaptic plasticity, gene therapy that targets pathogenic mutations or improves neurotrophic signaling holds potential in HD, AD and PD [133,134]. ASO-based treatments that show possibility for mutation-dependent intervention, like Tofersen for SOD1-ALS, have progressed to advanced-stage trials [135,136]. The goal of immunotherapies targeting pathogenic proteins is to minimize neurotoxicity and aggregation [137,138]. Early clinical evaluation shows that certain natural substances and small chemicals like PDE inhibitors have neuroprotective and anti-inflammatory properties [139,140]. Recent evidence has also identified Trace Amine-Associated Receptor 1 (TAAR1) as a promising therapeutic target in neurodegenerative disorders. TAAR1 regulates glutamatergic and monoaminergic neurotransmission, and its activation has been associated with improved synaptic plasticity, modulation of NMDA receptor function, and neuroprotective effects. Conversely, impaired TAAR1 signaling may contribute to glutamate excitotoxicity, mitochondrial dysfunction, and progressive neuronal damage, highlighting the therapeutic potential of TAAR1 agonists in NDs [141]. Simultaneously, biomarker-guided trial designs and iPSC-based tailored platforms improve therapy precision and enable patient stratification [142,143]. A summary of emerging therapeutic approaches and their clinical development status in neurodegenerative diseases is presented in Table 3.

## 5. Artificial Intelligence in Neurodegeneration

AI has become a revolutionary tool in ND research owing to the complexity of these disorders. They are multifactorial diseases, which complicate evaluation, therapy and monitoring. AI facilitates the integration of multimodal data, including neuroimaging, genetics and clinical aspects to uncover hidden patterns that expedite early diagnosis and alternative therapies [144,145]. Advanced machine learning algorithms used for MRI, PET and CT scans can detect cortical thinning, hippocampus atrophy and dopaminergic deficiencies more sensitively than conventional techniques. Convolutional neural networks have shown an advanced level of efficacy in predicting the progression from mild cognitive impairment to AD [146,147]. As AI use in diagnostic research grows, computational biomarkers obtained from speech, motion and writing analysis are being investigated more and more for early PD detection in addition to imaging [148,149].

AI is also widely used in patient monitoring and disease progression modeling. Precision medicine strategies can be guided by predictive algorithms that estimate individual trajectories for mental or motor decline by combining clinical, molecular, and imaging data [150]. Wearables with AI and Internet of Things gadgets enable continuous monitoring of behavioral, mobility, and sleep factors, generating real-time statistics that improve therapeutic decision-making [151]. Tools for natural language processing are being additionally modified to improve the digital phenotyping of NDs by identifying linguistic and facial recognition deficiencies [149]. Bibliometric analysis also shows that since 2000, the use of AI in NDs has grown significantly, reflecting both clinical necessity and scholarly interest [152].

AI in healthcare accelerates drug research and optimization by repositioning options and searching through vast bioinformatics and genomic information for new targets [153]. Deep learning-powered chemical modeling accelerates the discovery of potential drugs with desired safety and pharmacokinetic profiles compared to conventional pipelines. In a similar vein, neuromodulation methods such as deep brain and transcranial magnetic stimulation are being enhanced by AI-driven adaptive procedures to improve patient outcomes and adjust stimulation parameters [154]. The foundation for hybrid diagnosis–treatment systems is being laid by editorial perspectives that highlight how AI is increasingly collaborating with biosensing and brain stimulation procedures [155]. The power of AI is still limited by challenges such as annotated datasets, population biases, and cross-cohort generalization [156]. There are concerns about data privacy, and many AI systems work like black boxes (making it difficult to understand how decisions are made, which reduces trust in healthcare) [151,157]. Despite these challenges, AI is transforming ND research through rapid drug development, enabling early detection, enhancing patient monitoring, and enabling therapeutic options [158,159].

## 6. Conclusions and Future Perspectives

Neurodegenerative disorders are characterized by complex, system-level failures driven by the convergence of metabolic stress, immune dysregulation, and impaired proteostasis. The intricate crosstalk among HIF-1α, Notch, and JAK/STAT pathways emerges as a fundamental regulatory hub that determines the balance between neuronal adaptation and degeneration. Persistent activation of this signaling triad sustains a self-amplifying cycle of inflammation, mitochondrial dysfunction, and synaptic failure, explaining the inadequate success of single-target therapeutic strategies. Future research must therefore shift toward multi-target and systems-based approaches that integrate molecular, cellular, and environmental factors. Advances in biomarkers and artificial intelligence-driven analytics offer promising avenues for early diagnosis, patient stratification, and precision therapeutics. Ultimately, targeting the dynamic interplay between these pathways may yield more effective strategies to halt, or even reverse, neurodegenerative progression.

## Figures and Tables

**Table 1:** Details of chaperone with their mechanism of action, preclinical and clinical data.

S. No.	Chaperone	Primary Target Protein	Mechanism of Action	Associated Disease	Preclinical/Clinical Evidence	Key Findings	Reference
1	Tafamidis	Transthyretin (TTR)	Stabilizes native TTR tetramer, prevents monomer dissociation and β-sheet amyloid nucleation	ATTR amyloidosis	Human cardiomyocytes, mouse ATTR models: reduced fibril burden, improved survival/FDA Approved	Demonstrates that kinetic stabilization of native fold prevents amyloidogenesis	[32]
2	Hsp70	TDP-43, FUS	Binds disordered regions, maintains proteins in liquid-like phase, blocks amyloid nucleation	ALS, FTD	Cell and in vitro phase-separation models: prevents transition to fibrils	Directly suppresses amyloid conversion during LLPS	[33,34]
3	Hsp90	TDP-43	Controls folding trajectory; excessive activity stabilizes toxic conformers	ALS	*C. elegans* ALS model: reduced neurotoxicity upon Hsp90 inhibition	Lower Hsp90 activity re-routes misfolded TDP-43 toward clearance	[35]
4	Grp78/BiP (HspA5)	TDP-43	Direct binding suppresses aggregation-prone conformations	ALS	Neuronal cultures: reduced cytotoxicity and aggregate load	ER chaperones act as frontline proteostasis buffers	[36]
5	Hsp70–Hsp90 system	Tau, α-synuclein, TDP-43	Balances refolding vs. degradation; modulates energy landscape of misfolded states	AD, PD, ALS	Yeast and mammalian models: altered aggregation kinetics	“Folding vs. holding” determines fate of misfolded species	[37]
6	Small Heat Shock Proteins (sHSPs)	Aβ, α-synuclein, Tau	Bind exposed hydrophobic patches; inhibit β-sheet stacking	AD, PD	In vitro amyloid assays: suppressed fibril elongation	Act as kinetic traps preventing nucleation	[38]
7	Hsp70-based nanomotors	α-Synuclein	Targeted delivery + chaperone-mediated disaggregation	PD	Mouse PD model: reduced Lewy-like inclusions	Demonstrates active, spatially controlled proteostasis repair	[39]
8	Extracellular chaperones (Clusterin)	Aβ, Tau	Bind misfolded extracellular species, prevent seeding and spread	AD	CSF and mouse studies: reduced plaque propagation	Extends chaperone concept beyond intracellular space	[40]
9	Chemical chaperones (4-PBA, TUDCA)	Misfolded ER proteins	Reduce ER stress, stabilize folding intermediates	AD, PD	Rodent models: restored proteostasis, reduced apoptosis	Indirectly reshape folding landscape	[41]
10	Proteostasis network modulators	Multiple amyloidogenic proteins	Reprogram folding–degradation equilibrium	Multi-ND	Cellular models: enhanced clearance flux	Targets folding chemistry at network scale	[29,42]

**Table 2:** FDA-approved treatments for neurodegenerative diseases.

S. No.	Disease	Drug Name	Mechanism of Action	Therapeutic Target	FDA Approval Year	Reference
1	Alzheimer’s disease	Donepezil	Acetylcholinesterase inhibition	Cholinergic neurons	1996	[125]
2		Rivastigmine	Acetyl- and butyrylcholinesterase inhibition	Cholinergic neurons	2000	
3		Galantamine	Acetylcholinesterase inhibition, nicotinic modulation	Cholinergic neurons	2001	
4		Memantine	NMDA receptor antagonism	Glutamatergic signaling	2003	
5		Aducanumab	Monoclonal antibody clearing Aβ	Amyloid plaques	2021	[126]
6		Lecanemab	Monoclonal antibody, soluble Aβ protofibril binding	Amyloid plaques	2023	
7	Parkinson’s disease	Levodopa/Carbidopa	Dopamine precursor + DDC inhibition	Nigrostriatal dopamine	1970	[127]
8		Pramipexole	Dopamine D2/D3 receptor agonist	Dopaminergic pathway	1997	
9		Selegiline	Irreversible MAO-B inhibitor	Dopamine metabolism	1989	
10		Entacapone	COMT inhibitor, prolongs levodopa effect	Dopamine catabolism	1999	
11	Multiple sclerosis	Interferon-β	Cytokine modulation of T-cell activity	Immune regulation	1993	[129]
12		Glatiramer acetate	T-cell modulation, induction of Th2 cells	Immune regulation	1996	
13		Fingolimod	S1P receptor modulation, lymphocyte sequestration	Lymphocytes	2010	
14		Ocrelizumab	Anti-CD20 monoclonal antibody, B-cell depletion	B-cells	2017	
15	ALS	Riluzole	Glutamate release inhibition	Excitotoxicity	1995	[130]
16		Edaravone	Free radical scavenging	Oxidative stress	2017	
17		Sodium phenylbutyrate/taurursodiol (Relyvrio)	Mitochondrial and ER stress reduction	Mitochondrial function	2022	

**Table 3:** Emerging therapeutic targets and approaches.

S. No.	Target/Approach	Disease	Current Research Phase	Expected Outcome	Novel Therapeutic Targets	Reference
1	Gene therapy (AAV-mediated delivery)	AD, PD	Phase I/II	Enhance neurotrophic support and reduce pathology	NGF, GDNF, ApoE4 modulation	[133,134]
2	Antisense oligonucleotides (ASOs)	ALS	Phase III	Reduce toxic SOD1 protein expression	SOD1, C9orf72	[135,136]
3	Immunotherapy (anti-tau, anti-α-synuclein mAbs)	AD, PD, Tauopathies	Phase II/III	Halt aggregation and propagation	Tau, α-synuclein	[137,138]
4	PDE inhibitor (Ibudilast)	ALS, MS	Phase II	Neuroprotection, reduced inflammation	PDE4, PDE10	[140]
5	iPSC-derived cell therapy	AD, PD	Preclinical/Early Trials	Neuronal replacement, disease modeling	iPSC-derived dopaminergic neurons	[143]
6	Natural compounds (polyphenols, curcumin)	AD, HD	Phase I/II	Reduce oxidative stress and amyloid load	Polyphenol-responsive pathways	[139]
7	Biomarker-guided trial design	AD, ALS, PD	Ongoing clinical strategy	Improve stratification and therapeutic response	Fluid biomarkers (NfL, pTau), imaging markers	[142]

## Data Availability

The information supporting this study’s findings is available in this article.
